# The Financial Burden of Surgery for Congenital Malformations—The Austrian Perspective

**DOI:** 10.3390/ijerph182111166

**Published:** 2021-10-24

**Authors:** Paolo Gasparella, Georg Singer, Bernhard Kienesberger, Christoph Arneitz, Gerhard Fülöp, Christoph Castellani, Holger Till, Johannes Schalamon

**Affiliations:** 1 Department of Paediatric and Adolescent Surgery, Medical University of Graz, 8036 Graz, Austria; paolo.gasparella@medunigraz.at (P.G.); georg.singer@medunigraz.at (G.S.); bernhard.kienesberger@medunigraz.at (B.K.); c.arneitz@medunigraz.at (C.A.); christoph.castellani@medunigraz.at (C.C.); holger.till@medunigraz.at (H.T.); 2 Austrian Society of Paediatric and Adolescent Surgery, 1010 Vienna, Austria; 3 Austrian National Public Health Institute (Gesundheit Österreich GmbH, GÖG), 1010 Vienna, Austria; gerhard.fueloep@goeg.at

**Keywords:** congenital malformations, rare disease, neonatal surgery, health system

## Abstract

Neonatal “surgical” malformations are associated with higher costs than major “non-surgical” birth defects. We aimed to analyze the financial burden on the Austrian health system of five congenital malformations requiring timely postnatal surgery. The database of the Austrian National Public Health Institute for the period from 2002 to 2014 was reviewed. Diagnosis-related group (DRG) points assigned to hospital admissions containing five congenital malformations coded as principal diagnosis (esophageal atresia, duodenal atresia, congenital diaphragmatic hernia, gastroschisis, and omphalocele) were collected and compared to all hospitalizations for other reasons. Out of 3,518,625 total hospitalizations, there were 1664 admissions of patients with the selected malformations. The annual mean number was 128 (SD 17, range 110–175). The mean cost of the congenital malformations per hospital admission expressed in DRG points was 26,588 (range 0–465,772, SD 40,702) and was significantly higher compared to the other hospitalizations (*n* = 3,516,961; mean DRG 2194, range 0–834,997; SD 6161; *p* < 0.05). Surgical conditions requiring timely postnatal surgery place a significant financial burden on the healthcare system. The creation of a dedicated national register could allow for better planning of resource allocation, for improving the outcome of affected children, and for optimizing costs.

## 1. Introduction

Congenital malformations affect 3% of newborns in the United States and are responsible for 20% of deaths in the first year of life [1,2,3]. Only a limited number of patients born with malformations require timely surgical correction of the malformation after birth. This includes patients with esophageal atresia with or without tracheoesophageal fistula (EA/TEF), duodenal atresia (DA), congenital diaphragmatic hernia (CDH), gastroschisis (GS), and omphalocele (OM). With a prevalence of less than 2 per 2000, all the above-mentioned malformations belong to the group of rare diseases [4]. The European Commission recently established supranational networks of specialists and patients advocates, the so-called European Reference Networks (ERNs), aimed at improving treatment, allocating resources in a targeted way, and promoting research in this field [5].

In order to achieve the best possible outcome, the small number of patients born with congenital malformation require a conspicuous provision of resources, not only for initial treatment, but also for long-term follow-up. Furthermore, the quality of the initial treatment could potentially affect both the patients’ future quality of life and the subsequent costs of lifelong care [6]. This relationship is particularly well known for congenital heart diseases. A recent review of the long-term follow-ups of 228 patients revealed the major economic burden of these conditions [7].

It has recently been highlighted that “surgical” malformations are associated with higher costs than major “non-surgical” birth defects. This holds especially true for preterm babies [1]. Furthermore, it is a matter of vivid discussion whether a high volume of patients treated in a single center would ensure a better level of care and, therefore, could impact a patient’s outcome [8]. The relationship between high-volume centers, improved outcome, and costs reduction has already been demonstrated for other conditions. A large review including patients with congenital heart malformations has revealed significantly decreased hospital costs and more efficient resource allocation for patients admitted to intensive care units specializing in cardiac care [9]. This centralization also appears necessary to allow for better follow-up and optimal management of complications [10]. Despite the growing interest in understanding the costs of each one of the above-mentioned congenital malformations, data concerning the impact of these diseases on the Austrian health system are lacking [11].

Currently, about 8.8 million people live in Austria and the health system is entrusted to the nine federal states of the Austrian federation. In this territory, seven pediatric surgical departments meet the needs of the majority of the population.

The aim of this study was to analyze the financial burden on the healthcare system of five rare congenital malformations requiring timely postnatal surgery.

## 2. Materials and Methods

The database of the Austrian National Public Health Institute (Gesundheit Österreich GmbH, GÖG), an institution responsible for researching and planning public healthcare in Austria, was retrospectively reviewed.

Since 1997, the estimated costs of the Austrian national health system are calculated on the basis of the so-called DRG model (diagnosis-related groups). This system is based on the attribution of points according to standardized parameters at the national level. These include the medical procedures performed, the illnesses found, or the diagnosis coded via ICD-10, the age, and the hospital departments involved. The DRG model represents a powerful tool to standardize the quantification of expended and needed resources. A corresponding value in EUR to a single dataset is, however, not possible [12].

Data about the number of DRG points of each single admission in the general acute care and non-rehabilitation hospitals were collected. Due to a change in the electronic documentation system, data availability with respect to the research question was most adequate in the period from 2002 to 2014. Therefore, the present publication is based on this time frame.

For the present study, we collected the number of DRG points assigned to hospital admissions containing one of the following malformations coded as principal diagnosis: ICD-9-CM Q39.0 or Q39.1 (EA/TEF), Q41.0 (DA), Q79.0 (CDH), Q79.3 (GA) and Q79.2 (OM). These 5 neonatal surgical conditions are usually coded as principal diagnosis exclusively after birth, while in cases of subsequent hospitalizations, the principal diagnosis corresponds to the complication and the primary malformation is coded as secondary diagnosis. For this reason, the number of hospitalizations with the surgical anomaly coded as principal diagnosis roughly reflects the number of patients affected. We did not consider hospitalizations when the malformation did not appear as principal diagnosis.

The DRG points of each hospitalization for the considered anomalies and those of each single hospitalization for any other diagnosis during the period from 2002 to 2014 were collected.

The present study was approved by the local Ethics Committee (EK 33-239 ex 20-21).

Statistical analysis was performed using IBM^®^ SPSS^®^ Statistics, version 22 (IBM Corp., Armonk, NY, USA). Data are presented as means, ranges and standard deviations (SD). Statistical comparisons of DRG points between congenital malformations and other hospitalizations were performed with a Mann–Whitney U test. Comparison of the DRG points per admission of the five congenital malformations was performed with a Kruskal-Wallis test. Post hoc analysis with Mann–Whitney U tests was conducted with a Bonferroni correction applied. A *p*-value of <0.05 was considered statistically significant.

## 3. Results

### 3.1. Total Hospitalizations

**Figure 1:**
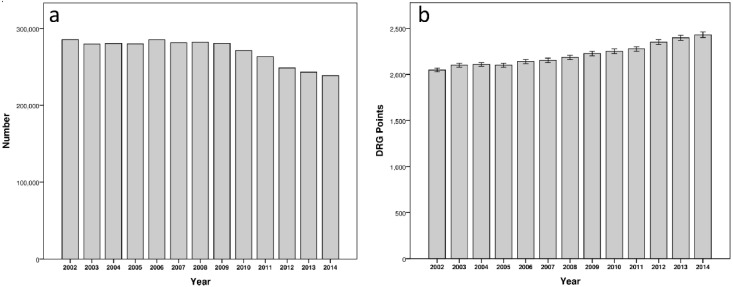
(**a**) The annual number of hospitalizations showed a decreasing trend in the period considered; (**b**) on the contrary the costs expressed in DRG points per admission increased over time.

During the 13-year study period, 3,518,625 hospitalizations of patients aged under 19 years were recorded in the Austrian healthcare system. The annual mean number of hospitalizations was 270,663 (SD 16,744, range 238,433–285,455). Over the years, there was a steady decrease in the number of hospitalizations from 285,455 in 2002 to 238,433 in 2014 (Figure 1a). The mean cost per hospital admission expressed in DRG points in the same period was 2205. The basis of this mean was all types of conditions requiring hospital admission and, therefore, there was a wide range between 0 and 834,997 DRG points (SD 6245). During the study period, there was a steady increase in DRG points per admission, starting from a mean of 2047 in 2002 rising to a mean of 2429 in 2014 (Figure 1b).

### 3.2. Congenital Malformations and Comparison to Other Hospitalizations

**Figure 2:**
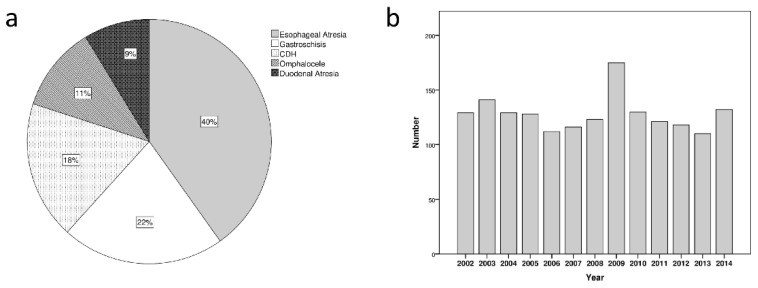
(**a**) In the pie chart the distribution of the five malformations considered is represented. (**b**) The number of hospitalizations for the malformations considered remained similar over the examined years.

Out of the 3,518,625 total hospitalizations, there were 1664 admissions of patients with the studied congenital malformations in the 13 years analyzed. The distribution of the five congenital malformations is depicted in Figure 2a. The annual mean number was 128 (range 110–175, SD 17) and we saw a relatively even distribution between 2002 and 2014 (Figure 2b).

The distribution of the malformations in the individual years is shown in Table 1. In detail, EA/TEF was registered for 668 hospitalizations (mean per year 51; range 40–63; SD: 5.9), followed by GS with 359 (mean per year 27; range 16–37; SD 5.7), CDH with 304 (mean per year 23; range 16–41; SD: 6.7), OM with 188 (mean per year 14; range 6–21; SD: 5.2), and DA with 145 (mean per year 10; range 4–22; SD: 4.8) (Table 1).

The mean cost per hospital admission expressed in DRG points of the congenital malformations was 26,588 (range 0–465,772, SD 40,702) and was significantly higher compared to the amount of DRG points of all the other hospitalizations together (*n* = 3,516,961; mean DRG 2194, range 0–834,997; SD 6161; *p* < 0.05). In detail, GS cost almost 15.61 times more than all other admissions. The burden for the remaining conditions was approximately 13.91 times higher for CDH, 14.97 for OM, 10.50 for DA and 8.82 for EA/TEF respectively (Table 2).

**Figure 3:**
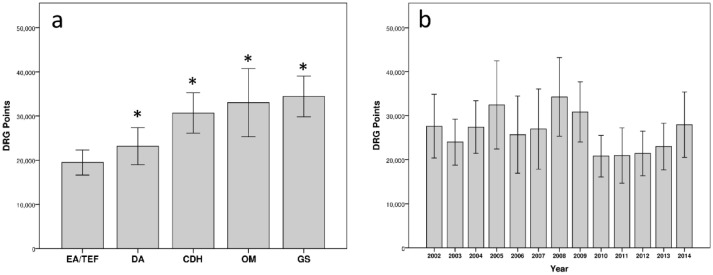
(**a**) Mean DRG points of the five selected malformations, the values for EA/TEF were significantly lower than those for all other malformations (* *p* < 0.05 vs. EA/TEF), abdominal wall defects represented the most expensive malformations. (**b**) Over the years, there was relatively even distribution of mean DRG points for all malformations together.

When comparing the DRG points of the five congenital malformations, EA was associated with a significantly lower number of DRG points per admission than DA, CDH, OM and GS (Figure 3a). Over the years, there were no statistically significant changes of DRG points per admission of the five different malformations (Figure 3b).

The DRG points of the five different malformations and the other malformations for the years 2002 to 2014 are presented in Supplementary Table S1.

## 4. Discussion

With this study, we demonstrated how five different congenital malformations, which usually require timely surgery after birth, influence the budget of the national health system in Austria. In particular, we found costs ranging between 8.82 times higher for esophageal atresia to 15.61 times higher for gastroschisis compared to all other hospitalizations.

Rare diseases are conditions with a prevalence of less than 1 per 2000 [5]. Among others, congenital malformations represent a large part of these diseases [13]. The five congenital malformations included in our analysis have low incidences of 1 out of 2500 newborns for EA, 1–2 out of 10,000 for DA, 1–5 out of 10,000 for CDH, 1 out of 5000 for GS and 1 out of 8500 for OM. Despite their low incidence, it is increasingly recognized that the financial and clinical burden of these congenital malformations is not negligible. Therefore, there is a growing interest to optimize associated costs [14,15,16]. In a recent analysis of the North-Eastern Italian Registry, rare diseases had an overall incidence of 3.85 per 10,000 inhabitants. Among these, pediatric congenital malformations were recognized as a relevant public health issue [17].

In the past, the surgeon acted as the main player in the treatment of these pathologies and surgery was considered the only variable influencing their prognosis. Over the years, however, awareness of the complexity of these malformations has developed and the necessity of diverse and dedicated healthcare professionals to care for these patients has been recognized. The higher costs of admissions for patients with GS, for example, probably reflects the length of the postnatal hospital stay due to the time these patients need for weaning from parenteral nutrition [18]. Furthermore, complex GS in particular can be associated with complications, such as prolonged ileus, sepsis, wound infection, and necrotizing enterocolitis, requiring multiple transfers to the intensive care unit and sometimes even surgical re-interventions. During this long hospitalization, pediatric intensivists, dieticians, nutritionists, gastroenterologists, as well as pediatric surgeons, are indispensable. Therefore, the single operation does not explain the burden of the costs of hospitalization for the different malformations studied.

Furthermore, these anomalies could present with associated anomalies necessitating a multidisciplinary experienced team. If EA is, for example, part of a VACTERL syndrome (vertebral abnormalities, anal atresia, cardiac defects, tracheo-esophageal abnormalities, renal anomalies, and limb abnormalities), other experts beside the pediatric surgeons are desirable for care, including cardiologists, otolaryngologists, orthopedic surgeons, as well as dedicated nursing staff [19].

Our data clearly show the financial impact of these diseases, which requires adequate staff preparation and facility planning. For this reason, centers aiming to treat these patients should have multidisciplinary teams experienced with the specific and multiple problems of these patients [20].

In a recent report, Schmedding and coworkers analyzed the outcome of patients who underwent pediatric surgical treatment for abdominal wall defects in the decentralized care system of Germany. The authors found mortality rates similar to that reported in the literature and deduced that the volume of the center does not influence patient outcome [21]. Nevertheless, other studies including similar conditions have demonstrated the opposite [11,22,23]. In our opinion, mortality as the only parameter for evaluating the effectiveness of the treatment of pathologies with such a degree of complexity is not sufficient. Furthermore, the question arises whether it is justifiable to keep many low-volume centers rather than allocating resources to optimize high-volume centers.

The referral to centers of reference should ideally take place already in the prenatal period [24]. Prenatal diagnosis of the majority of the five studied malformations is possible. Only EA/TEF often remains unnoticed despite the improvement of diagnostic tools [25]. The high level of experience and competence of reference centers should guarantee a better treatment of patients as well as an optimization of associated costs. Centralization of patients is also essential during long-term follow-up. Dedicated centers can guarantee standardized protocols and the early recognition of complications specific for each malformation [20].

Understanding the financial burden of the studied pathologies is an essential first step in raising the awareness of political and administrative leaders but must be followed by a proposal to improve planning. Therefore, it is of uppermost importance to obtain more accurate information about these pathologies. The creation of dedicated registers allowing the sharing of data concerning all aspects of rare diseases has proven to be the most appropriate start [26]. A structured registry also helps to facilitate the development of dedicated research on the basis of higher numbers, which by definition are lacking on rare diseases. For example, centralized collection of information on gastroschisis patients could be useful in understanding whether the time to achieve full enteral nutrition is different in high-volume versus low-volume centers, thereby allowing stakeholders to ultimately allocate more resources to one center rather than the other. Furthermore, if introduced and guided centrally, it could help to overcome the individualism of single centers. The effort in this direction by the European Union with the creation of the ERNs is evident. This process will foster an integration of experiences and efforts in treatment and research in the field of rare diseases [27].

In our opinion, it is necessary to raise the consciousness of politicians and stakeholders that, despite their low incidence, the examined congenital malformations represent an important part of resource allocation. The examined malformations are rare, but their treatment is costly. Moreover, considering our results, we suggest creating dedicated registers and to implement centers with proved expertise in treating rare conditions in order to facilitate measurement and comparison of outcomes.

Important limitations of our study are the impossibility of correlating the single diagnosis to a specific patient and the non-direct convertibility of the DRG points to a corresponding EUR value. Consequently, it was not possible to correlate these costs with other important indicators, such as the duration of hospitalization, comorbidity, frequency of complications, and mortality. However, the comparison between DRG points invested in different pathologies stratified by year allows an accurate examination of the additional financial burden determined by the considered anomalies. To the best of our knowledge, our data are the first comparing the costs of hospitalizations for congenital malformations to that of all other diagnoses.

## 5. Conclusions

The considered congenital malformations requiring timely surgery after birth represent a significant financial burden in Austria. The actual lack of available information prevents improvement in treatment planning for these rare diseases. Therefore, the prospective creation of dedicated registers could implement the possibility of sharing information, comparing outcomes, and consequently improve the quality of care and research in the field of rare congenital malformations. These measures may also result in a decrease in costs. Furthermore, the involvement of patient organizations could improve the future organization of treatment paths in order to provide optimal care for our patients.

## Figures and Tables

**Table 1:** Number of hospitalizations by year and considered congenital malformations (EA/TEF: esophageal atresia; DA: duodenal atresia; CDH: congenital diaphragmatic hernia; OM: omphalocele; GS: gastroschisis).

Year	Other Diagnoses	EA/TEF	DA	CDH	OM	GS	Total Malformations	Total Admissions
2002	285,326	48	12	17	21	31	129	285,455
2003	279,458	51	22	24	11	33	141	279,599
2004	280,181	48	9	27	16	29	129	280,310
2005	279,759	49	9	23	20	27	128	279,887
2006	285,162	56	10	18	6	22	112	285,274
2007	281,191	54	9	17	14	22	116	281,307
2008	281,938	51	4	29	13	26	123	282,061
2009	280,322	63	19	41	21	31	175	280,497
2010	270,862	59	10	21	6	34	130	270,992
2011	263,109	53	6	26	10	26	121	263,230
2012	248,401	51	12	16	14	25	118	248,519
2013	242,951	45	10	21	18	16	110	243,061
2014	238,301	40	13	24	18	37	132	238,433
	3,516,961	668	145	304	188	359	1664	3,518,625

**Table 2:** Costs as expressed in DRG points per hospital admission and factor comparing the five selected congenital malformations to all other hospitalizations aged 0 to 19 years.

	DRG Points [Mean ± SD]	Factor
Other Hospitalizations	2205 ± 6245	1
EA/TEF	19,454 ± 36,761	8.82
DA	23,161 ± 25,395	10.50
CDH	30,668 ± 39,915	13.91
OM	33,015 ± 52,983	14.97
GS	34,426 ± 43,663	15.61

## Data Availability

The data presented in this study are available on request from the corresponding author.

## Supplementary Materials

The following are available online at https://www.mdpi.com/article/10.3390/ijerph182111166/s1, Table S1: DRG points per hospital admission because of the selected congenital malformations in the years 2002 to 2014; data are presented as mean ± SD.

## References

[1] Apfeld J.C., Kastenberg Z.J., Gibbons A.T., Phibbs C.S., Lee H.C., Sylvester K.G. (2019). The disproportionate cost of operation and congenital anomalies in infancy. Surgery.

[2] Arth A.C., Tinker S.C., Simeone R.M., Ailes E.C., Cragan J.D., Grosse S.D. (2017). Inpatient Hospitalization Costs Associated with Birth Defects Among Persons of All Ages—United States, 2013. MMWR Morb. Mortal. Wkly. Rep..

[3] Higashi H., Barendregt J.J., Kassebaum N.J., Weiser T.G., Bickler S.W., Vos T. (2015). The burden of selected congenital anomalies amenable to surgery in low and middle-income regions: Cleft lip and palate, congenital heart anomalies and neural tube defects. Arch. Dis. Child..

[4] Richter T., Nestler-Parr S., Babela R., Khan Z.M., Tesoro T., Molsen E., Hughes D.A., International Society for Pharmacoeconomic, Outcomes Research Rare Disease Special Interest Group (2015). Rare Disease Terminology and Definitions—A Systematic Global Review: Report of the ISPOR Rare Disease Special Interest Group. Value Health.

[5] Cannizzo S., Lorenzoni V., Palla I., Pirri S., Trieste L., Triulzi I., Turchetti G. (2018). Rare diseases under different levels of economic analysis: Current activities, challenges and perspectives. RMD Open.

[6] Arnold H.E., Baxter K.J., Short H.L., Travers C., Bhatia A., Durham M.M., Raval M.V. (2018). Short-term and family-reported long-term outcomes of simple versus complicated gastroschisis. J. Surg. Res..

[7] Lee V.W.Y., Yan B.P., Fong T.M.C., Fung A.K.P., Cheng F.W.T. (2019). Long-term health-related burden of adult congenital heart diseases in Hong Kong. J. Med. Econ..

[8] Apfeld J.C., Kastenberg Z.J., Sylvester K.G., Lee H.C. (2017). The Effect of Level of Care on Gastroschisis Outcomes. J. Pediatr..

[9] Johnson J.T., Wilkes J.F., Menon S.C., Tani L.Y., Weng H.Y., Marino B.S., Pinto N.M. (2018). Admission to dedicated pediatric cardiac intensive care units is associated with decreased resource use in neonatal cardiac surgery. J. Thorac. Cardiovasc. Surg..

[10] Ronnekleiv-Kelly S.M., Soares K.C., Ejaz A., Pawlik T.M. (2016). Management of choledochal cysts. Curr. Opin. Gastroenterol..

[11] Somme S., Shahi N., McLeod L., Torok M., McManus B., Ziegler M.M. (2019). Neonatal surgery in low- vs. high-volume institutions: A KID inpatient database outcomes and cost study after repair of congenital diaphragmatic hernia, esophageal atresia, and gastroschisis. Pediatr. Surg. Int..

[12] Hagenbichler E. (2010). The Austrian DRG System.

[13] Ferreira C.R. (2019). The burden of rare diseases. Am. J. Med. Genet. A.

[14] Austin C.P., Cutillo C.M., Lau L.P.L., Jonker A.H., Rath A., Julkowska D., Thomson D., Terry S.F., de Montleau B., Ardigo D. (2018). Future of Rare Diseases Research 2017–2027: An IRDiRC Perspective. Clin. Transl. Sci..

[15] Dingemann C., Eaton S., Aksnes G., Bagolan P., Cross K.M., De Coppi P., Fruithof J., Gamba P., Husby S., Koivusalo A. (2020). ERNICA Consensus Conference on the Management of Patients with Esophageal Atresia and Tracheoesophageal Fistula: Diagnostics, Preoperative, Operative, and Postoperative Management. Eur. J. Pediatr. Surg..

[16] Groft S.C., Posada de la Paz M. (2017). Preparing for the Future of Rare Diseases. Adv. Exp. Med. Biol..

[17] Mazzucato M., Visona Dalla Pozza L., Manea S., Minichiello C., Facchin P. (2014). A population-based registry as a source of health indicators for rare diseases: The ten-year experience of the Veneto Region’s rare diseases registry. Orphanet J. Rare Dis..

[18] Bradnock T.J., Marven S., Owen A., Johnson P., Kurinczuk J.J., Spark P., Draper E.S., Knight M., Baps C. (2011). Gastroschisis: One year outcomes from national cohort study. BMJ.

[19] Shetty S., Kennea N., Desai P., Giuliani S., Richards J. (2016). Length of stay and cost analysis of neonates undergoing surgery at a tertiary neonatal unit in England. Ann. R. Coll. Surg. Engl..

[20] Jsselstijn H., Gischler S.J., Wijnen R.M.H., Tibboel D. (2017). Assessment and significance of long-term outcomes in pediatric surgery. Semin. Pediatr. Surg..

[21] Schmedding A., Wittekind B., Salzmann-Manrique E., Schloesser R., Rolle U. (2020). Decentralized surgery of abdominal wall defects in Germany. Pediatr. Surg. Int..

[22] Dubrovsky G., Sacks G.D., Friedlander S., Lee S. (2017). Understanding the relationship between hospital volume and patient outcomes for infants with gastroschisis. J. Pediatr. Surg..

[23] Youssef F., Cheong L.H., Emil S., The Canadian Pediatric Surgery Network (2016). Gastroschisis outcomes in North America: A comparison of Canada and the United States. J. Pediatr. Surg..

[24] Aite L., Zaccara A., Cuttini M., Mirante N., Nahom A., Bagolan P. (2013). Lack of institutional pathways for referral: Results of a survey among pediatric surgeons on prenatal consultation for congenital anomalies. Prenat. Diagn..

[25] Pardy C., D’Antonio F., Khalil A., Giuliani S. (2019). Prenatal detection of esophageal atresia: A systematic review and meta-analysis. Acta Obstet. Gynecol. Scand..

[26] Monaco L., Crimi M., Wang C.M. (2014). The challenge for a European network of biobanks for rare diseases taken up by RD-Connect. Pathobiology.

[27] Moliner A.M., Waligora J. (2017). The European Union Policy in the Field of Rare Diseases. Adv. Exp. Med. Biol..

