# Supplementary material for: The Financial Burden of Surgery for Congenital Malformations—The Austrian Perspective
Source: Int J Environ Res Public Health. 2021 Oct 24;18(21):11166. doi: 10.3390/ijerph182111166 (PMC8582705; doi:10.3390/ijerph182111166)
Supplement: Supplementary file 1 [file ijerph-18-11166-s001.zip › ijerph-1401252-SI.pdf]

Supplementary Table S1: DRG points per hospital admission because of the selected congenital malformations in the years 2002 to 2014; data are presented as mean ± SD.

Year	EA/TEF	DA	CDH	OM	GS
2002	34,424 ± 61,570	15,979 ± 10,827	19,833 ± 21,077	26,633 ± 15,484	26,508 ± 25,710
2003	17,449 ± 28,083	18,054 ± 11,620	24,795 ± 27,424	24,208 ± 22,833	37,488 ± 43,772
2004	24,647 ± 41,956	30,142 ± 43,054	28,090 ± 26,996	31,321 ± 27,636	28,300 ± 25,782
2005	23,416 ± 46,374	19,192 ± 12,681	26,463 ± 27,135	34,132 ± 45,357	57,172 ± 93,052
2006	19,215 ± 38,553	15,999 ± 12,589	22,919 ± 22,459	70,403 ± 148,935	36,630 ± 29,625
2007	19,068 ± 34,551	25,036 ± 18,385	23,321 ± 21,633	52,892 ± 117,702	33,465 ± 21,119
2008	18,267 ± 29,586	31,255 ± 5,676	56,124 ± 69,211	24,206 ± 21,088	46,810 ± 57,741
2009	19,171 ± 32,889	37,768 ± 43,584	40,026 ± 52,254	33,605 ± 44,212	36,366 ± 55,359
2010	11,410 ± 19,207	15,848 ± 13,160	26,812 ± 27,542	30,111 ± 24,540	33,221 ± 35,030
2011	14,565 ± 29,879	16,536 ± 16,697	24,960 ± 50,908	27,764 ± 36,219	28,251 ± 23,769
2012	10,975 ± 20,872	20,399 ± 14,441	33,444 ± 34,005	26,147 ± 30,439	32,867 ± 30,827
2013	22,266 ± 31,886	15,644 ± 17,165	21,685 ± 26,755	23,659 ± 26,591	30,616 ± 24,455
2014	21,762 ± 46,724	33,726 ± 37,383	30,979 ± 30,486	42,438 ± 63,801	23,610 ± 33,719
